# Association of Duration of Surgery With Postoperative Delirium Among Patients Receiving Hip Fracture Repair

**DOI:** 10.1001/jamanetworkopen.2019.0111

**Published:** 2019-02-22

**Authors:** Bheeshma Ravi, Daniel Pincus, Stephen Choi, Richard Jenkinson, David N. Wasserstein, Donald A. Redelmeier

**Affiliations:** 1 Division of Orthopaedic Surgery, Department of Surgery, University of Toronto, Toronto, Ontario, Canada; 2 Division of Orthopaedic Surgery, Sunnybrook Health Sciences Centre, Toronto, Ontario, Canada; 3 Institute for Clinical Evaluative Sciences, Toronto, Ontario, Canada; 4 Department of Anesthesia, Sunnybrook Health Sciences Centre, Toronto, Ontario, Canada; 5 Department of Medicine, University of Toronto, Toronto, Ontario, Canada

## Abstract

**Importance:**

Postoperative delirium in older adults receiving hip fracture surgery is associated with morbidity and increased health care costs, yet little is known of potential modifiable factors that may help limit the risks.

**Objective:**

To use population-wide individual-level data on the duration of hip fracture surgery to determine whether prolonged surgical times and type of anesthesia are associated with an increased risk of postoperative delirium.

**Design, Setting, and Participants:**

This retrospective population-based cohort study analyzed patients aged 65 years and older receiving hip fracture surgery between April 1, 2009, and March 30, 2017, at 80 hospitals in Ontario, Canada. Generalized estimated equations with logistic regression analysis were used to determine the relationship between procedure duration, type of anesthesia, and the occurrence of postoperative delirium. Restricted cubic splines were also generated to visualize this relationship. Data analysis was conducted from July to October 2018, revision in January 2019.

**Exposure:**

Surgery duration, measured as the total time in the operating room.

**Main Outcomes and Measures:**

A diagnosis of postoperative delirium during hospitalization.

**Results:**

Among 68 131 patients with surgically managed hip fracture (median [interquartile range] age, 84 [78-89] years; 72% women) identified, 7150 patients experienced postoperative delirium. In total, 26 853 patients (39.4%) received general anesthesia. Receiving general anesthesia was associated with a slightly higher rate of postoperative delirium compared with not receiving general anesthesia (2943 [11.0%] vs 4207 [10.2%]; *P* = .001). The risk for delirium increased with increased surgical duration—every 30-minute increase in the duration of surgery was associated with a 6% increase in the risk for delirium (adjusted odds ratio, 1.06; 95% CI, 1.03-1.08; *P* < .001). Prolonged surgical duration was associated with a higher incidence of postoperative delirium, and the risk was higher was in patients who had received general anesthesia (adjusted odds ratio, 1.08; 95% CI, 1.04-1.12; *P* < .001) than in those patients who did not receive GA (adjusted odds ratio, 1.04; 95% CI, 1.01-1.08; *P* = .01).

**Conclusions and Relevance:**

Among older adults receiving hip fracture surgery, both an increased duration of surgery and receiving a general anesthetic were associated with an increased risk for postoperative delirium.

## Key Points

**Question:**

Is surgery duration associated with postoperative delirium in patients receiving hip fracture repair?

**Findings:**

In this population-based cohort study of 68 131 adults, increasing surgery duration was associated with a higher risk-adjusted likelihood of postoperative delirium (6% increase in delirium risk per additional half hour of surgery). This risk was higher in patients who received a general anesthetic.

**Meaning:**

The findings suggest that prolonged surgery is associated with increased postoperative delirium, particularly when the patient has received a general anesthetic.

## Teaser

This population-based cohort study examines whether prolonged surgical times and type of anesthesia are associated with an increased risk of postoperative delirium among elderly patients with hip fracture.

## Introduction

Delirium is an acute change in mental status characterized by fluctuating disturbances of consciousness, attention, cognition, and perception.^1^ Elderly patients are prone to delirium after surgery^2^ as they often have major chronic comorbid conditions^3^ and a decreased physiological reserve to handle the stress of surgery.^4^ Delirium in elderly patients can contribute to significant postoperative morbidity and occasionally permanent disability.^5,6,7^ Unfortunately, risk modification that lower the risk for postoperative delirium is often not possible for urgent surgical procedures.

Hip fracture repair is the most common reason for urgent surgery in elderly patients, accounting for more than 300 000 hospital admissions in the United States annually.^8^ Elderly patients with hip fractures are particularly vulnerable to developing postoperative delirium, with an incidence ranging between 5% to 61%.^9,10^ In many cases, the hip fracture also signifies a premorbid decrease in function. A subsequent episode of perioperative delirium may result in a lifelong functional impairment, and increased mortality.^11,12,13^ Furthermore, delirium increases health care costs for the episode of care by more than 50%,^12^ owing to a combination of length of stay, extra nursing requirements, and intensified medical testing.^14,15,16^

The causes of postoperative delirium are complex and not well understood, and they include several preoperative and postoperative causes.^14,17,18^ Two underlying factors may be altered cerebral perfusion^19^ secondary to anesthesia^20^ and the body’s normal inflammatory response to surgery,^21^ of which both are exacerbated by prolonged surgical duration and may vary by type of anesthesia. The objective of our study was to assess the association between duration of hip fracture surgery and the risk for postoperative delirium in older adults. A secondary objective was to determine whether the route of anesthesia was associated with the risk for delirium.

## Methods

### Study Design and Data Sources

We conducted a population-based cohort study using administrative data from Ontario, Canada. Residents of Ontario were insured under a single-payer system that covered all medically necessary procedures including management and aftercare for hip fracture.^22,23,24,25^ The main data sources were hospital discharge abstracts from the Canadian Institute for Health Information Discharge Abstract Database, and physician service claims from the Ontario Health Insurance Plan Claims History Database, and the Registered Persons Databases.^24,26^ The study protocol was approved by the Research Ethics Board at Sunnybrook Health Sciences Centre, Toronto. Individual patient informed consent was not required for analyzing encoded administrative health data in Ontario. This study followed the Strengthening the Reporting of Observational Studies in Epidemiology (STROBE) reporting guideline.

### Patients

We identified patients who received acute surgical management for a hip fracture between April 1, 2009, and March 31, 2017. We excluded procedures in patients who were not from Ontario, those performed by nonorthopedic surgeons, and those performed in patients who died on or before the index date. We also excluded patients younger than age 65 years to limit the cohort to older adults. We excluded procedures shorter than 30 minutes or longer than 240 minutes, as these atypical cases are not representative of most hip fracture procedures.^27^

### Primary Exposure

The primary potential indicator was the duration of the surgical procedure. The start and end times for each procedure were defined as the entry and exit times of the operating room.^22^

### Additional Characteristics

Patient age and sex were obtained from the Discharge Abstract Database. Patient comorbidities were obtained from the 5 years before the index hospital admission and categorized according to an adaptation of the Deyo Charlson Comorbidity Index.^28^ Frail patients were identified using the John Hopkins Adjusted Clinical Groups indicator, based on diagnosis codes from hospitalizations and physician visits in the 5 years preceding the index hip fracture surgery.^29,30^ Preexisting dementia was identified from electronic medical records according to a validated algorithm (sensitivity: 79%, positive predictive value: 80%, negative predictive value: 99%).^31^ Median neighborhood household income quintile was used as a surrogate for socioeconomic status.^32,33,34^ Wait time for surgery was defined as the elapsed time from emergency department arrival until surgery in hours.^22^ Patient living location prior to hospitalization was obtained from the Discharge Abstract Database.

Index surgeon- and hospital-related factors were identified at time of each patient’s operation. Surgical volume was defined as the number of hip fracture procedures performed by the primary surgeon in the year preceding the index procedure.^35^ Hospitals were also categorized as either academic or community on the basis of their membership in the Council of Academic Hospitals of Ontario.^36^ In addition, use of general anesthesia (GA) for the hip fracture surgery was determined using a combination of the Discharge Abstract Database and billing codes from the Ontario Health Insurance Plan database.

### Primary Outcome

Our primary outcome was the occurrence of postoperative delirium during hospital admission. This diagnosis can be challenging to establish with hyperactive cases being more easily diagnosed.^37,38^ As such, there is a high level of ascertainment bias and potential for considerable variation between hospitals.^39,40^ The occurrence of postoperative delirium was identified using *International Statistical Classification of Diseases and Related Health Problems, Tenth Revision* codes for delirium, which have been shown to be highly specific but only weakly sensitive (specificity: 98%, sensitivity: 35%, positive predictive value: 100%).^14,41^ To account for variable coding between hospitals, we clustered by hospital in our regression analyses.

### Statistical Analysis

Baseline cohort characteristics were described using proportions and medians and were compared between groups using the Wilcoxon rank sum test for continuous variables and χ^2^ test for categorical variables. Generalized estimating equations were generated to determine the relationship between surgical duration and postoperative delirium after controlling for patient (age, sex, income quintile, and comorbidity) and hospital (teaching hospital and hospital volume) factors, type of anesthesia (GA or not), and clustering by the institution where the procedure was performed. Missing data (<1% for all variables considered) were excluded from regression models. We also generated restricted cubic splines (and 95% CIs) with 4 knots to model the probability of delirium and myocardial infarction according to the surgery duration. Cubic splines are mathematical functions that are composed of piecewise polynomial functions. Splines make no underlying assumptions about a functional form, and as such are useful to determine whether the relationship between 2 variables is nonlinear. Any nonlinear relationship can be assessed using spline regression. All analyses were performed using SAS, version 9.3 and SAS Enterprise Guide software, version 6.1 (SAS Institute Inc). A 2-tailed type I error probability was set to *P* < .05 for all analyses.

We repeated the generalized estimating equations models examining the relationship between surgical duration and postoperative delirium, controlling for the same factors, after stratifying our cohort by the type of anesthesia received (GA or not).

## Results

### Patient and Surgeon Characteristics

We identified 68 131 patients with surgically managed hip fractures between April 1, 2009, and March 31, 2017 (Table 1). The median age was 84 years (interquartile range, 78-89 years), 48 826 patients (71.7%) were female, 27 846 patients (40.9%) were categorized as frail. Almost three-quarters of patients were managed in community hospitals. Overall, 7150 patients were subsequently diagnosed with delirium.

**Table 1.** Selection of Patients for Inclusion

Cohort Selection	Patients, No.
Hip fracture surgery from April 1, 2009, to March 31, 2017	79 062
Exclusion criteria	
Not a resident of Ontario	88
Dead before or on index date	16
Surgery not performed by an orthopedic surgeon	396
Procedure <30 min or >240 min	1846
Age <65 y	8585
Final cohort	68 131

In total, 26 853 patients (39.4%) received GA (Table 2). Compared with patients who received a regional anesthetic, these patients were more likely to have had a femoral neck fracture (12 949 [48.2%]) and to have had surgery at a teaching hospital (8740 [32.5%]). Patients who had GA compared with patients who did not receive GA had a slightly higher rate of postoperative delirium (2943 [11.0%] vs 4207 [10.2%]; *P* = .001).

**Table 2.** Baseline Characteristics at the Time of Surgery, Stratified by Anesthesia Type

Cohort Characteristics	No. (%)		*P* Value
	General Anesthetic	No General Anesthetic	
No. of patients	26 853	41 278	
Age, median (IQR), y	84 (78-89)	84 (78-89)	<.001
Female	18 989 (70.7)	29 837 (72.3)	<.001
Income quintile			
Lowest	5812 (21.8)	8650 (21.0)	<.001
2	5505 (20.6)	8359 (20.3)	
3	5082 (19.1)	8387 (20.4)	
4	5114 (19.2)	8266 (20.1)	
Highest	5164 (19.4)	7441 (18.1)	
Dementia	5136 (19.1)	8044 (19.5)	.24
Frail	11 231 (41.8)	16 615 (40.3)	<.001
Charlson comorbidity index score			
0	15 382 (57.3)	25 482 (61.7)	<.001
1	4135 (15.4)	6090 (14.8)	
2	2655 (9.9)	3948 (9.6)	
≥3	4681 (17.4)	5758 (13.9)	
Prior location			
Acute care center	6097 (22.7)	7697 (18.6)	<.001
Chronic care center	6172 (23.0)	9652 (23.4)	
Unknown	14 584 (54.3)	23 929 (58.0)	
Teaching hospital	8740 (32.5)	10 023 (24.3)	<.001
Surgeon volume, median (IQR)	73 (38-111)	72 (39-109)	.08
Time from ED to OR, median (IQR), h	32 (22-50)	30 (21-48)	<.001
Duration of surgery, median (IQR), min	101 (80-129)	95 (76-120)	<.001
Fracture type			
Femoral neck	12 949 (48.2)	21 324 (51.7)	<.001
Intertrochanteric	12 286 (45.8)	17 934 (43.4)	
Subtrochanteric	1618 (6.0)	2020 (4.9)	
Fixation			
Cannulated screws	11 237 (41.8)	16 488 (39.9)	<.001
Hemiarthroplasty	10 050 (37.4)	17 513 (42.4)	
Intramedullary nail	5566 (20.7)	7277 (17.6)	
Calendar year			
2009	2329 (8.7)	3815 (9.2)	.004
2010	3106 (11.6)	5022 (12.2)	
2011	3265 (12.2)	4996 (12.1)	
2012	3270 (12.2)	5176 (12.5)	
2013	3517 (13.1)	5438 (13.2)	
2014	3594 (13.4)	5370 (13.0)	
2015	3566 (13.3)	5285 (12.8)	
2016	3621 (13.5)	5345 (12.9)	
2017	585 (2.2)	831 (2.0)	
Delirium	2943 (11.0)	4207 (10.2)	.001
Length of stay, median (IQR), d	8 (5-15)	8 (5-14)	<.001

Abbreviations: ED, emergency department; IQR, interquartile range; OR, operating room.

### Duration of Surgery and Postoperative Delirium

The restricted cubic spline curve suggested that increased duration of surgery (median [interquartile range], 101 [80-129] vs 95 [76-120] minutes) was associated with progressive increases in the occurrence of postoperative delirium (Figure). After controlling for patient age, sex, comorbidity (Charlson score, dementia, and frailty), income quintile, home location, type of anesthetic, and treatment factors (teaching hospital, delay to surgery, and GA), increased surgical duration was associated with an increased risk for delirium (adjusted odds ratio: 1.06 per additional 30 minutes of surgery; 95% CI, 1.03-1.08; *P* < .001) (Table 3). Prolonged surgical duration was associated with a higher incidence of postoperative delirium, and the risk was higher in patients who received a GA (adjusted odds ratio, 1.08; 95% CI, 1.04-1.12; *P* = .002) than in those patients who did not have a GA (adjusted odds ratio, 1.04; 95% CI, 1.01-1.08; *P* = .01).

**Figure.**
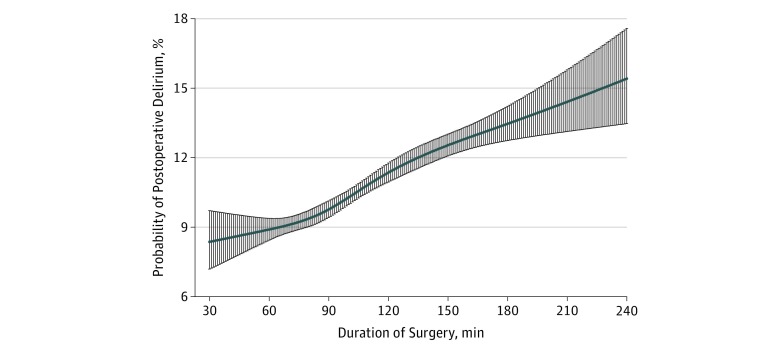
Probability of Postoperative Delirium (With 95% CI) vs Duration of Surgery

**Table 3.** Odds Ratio of Postoperative Delirum After Adjustment^a^

Characteristics	Adjusted OR (95% CI)	*P* Value
Age, y	1.04 (1.04-1.05)	<.001
Male	1.62 (1.54-1.70)	<.001
Income quintile		
Lowest	1.05 (0.97-1.14)	.20
2	1.01 (0.94-1.09)	.69
3	0.95 (0.88-1.03)	.24
4	0.99 (0.90-1.08)	.79
Highest	1 [Reference]	
Dementia	1.07 (0.97-1.17)	.18
Frail	1.72 (1.62-1.84)	<.001
Charlson comorbidity index score		
0	1 [Reference]	
1	1.04 (0.98-1.11)	.23
2	1.03 (0.96-1.10)	.45
≥3	1.08 (1.00-1.16)	.06
Prior location		
Acute care centre	0.86 (0.77-0.95)	.003
Chronic care centre	0.85 (0.77-0.93)	.001
Unknown	1 [Reference]	
Teaching hospital	1.58 (1.20-2.08)	.001
Time from ED to OR (per h)	1.01 (1.01-1.02)	.002
Fracture type		
Femoral neck	1 [Reference]	
Intertrochanteric	1.03 (0.95-1.11)	.51
Subtrochanteric	0.96 (0.85-1.08)	.49
Fixation		
Cannulated screws	1 [Reference]	
Hemiarthroplasty	1.12 (1.03-1.21)	.005
Intramedullary nail	1.00 (0.94-1.06)	.94
Calendar year (per year from 2009)	1.13 (1.10-1.16)	<.001
General anesthesia	1.07 (1.01-1.13)	.02
Length of procedure (per 30 min)	1.06 (1.03-1.08)	<.001

Abbreviations: ED, emergency department; OR, odds ratio; OR, operating room.

^a^ Adjusted for patient age, sex, income quintile, history of dementia, frailty, Charlson comorbidity, prior location, teach hospital, time from ED to OR, fracture type, fixation type, calendar year, type of anesthesia, and length of procedure.

## Discussion

In this population-based cohort study spanning the last 8 years in Ontario, Canada, approximately 11% of older adults who received hip fracture surgery were diagnosed with postoperative delirium. Both prolonged duration of surgery and GA were associated with an increased risk. Additional risk factors included increasing age, male sex, and patient frailty. Increased surgical duration remained probable of delirium after controlling for these factors, and this association was greater in patients who received a GA. Overall, every additional 30 minutes of surgery was associated with an approximate 6% relative increase in the risk for delirium after adjustment.

Postoperative delirium is a common complication following hip fracture surgery in older adults. Our results suggest that prolonged duration of surgery is associated with an increased risk for delirium. While the pathophysiology of postoperative delirium is multifactorial, a major factor may be disruptions of cerebral autoregulation that can occur during surgery.^53,54^ Specifically, hypercapnia, anemia, and hypothermia all contribute to diminished autoregulation and are exacerbated by prolonged surgery.^53,55,56^ Surgical duration is affected by many factors, including the complexity of the injury, the difficulty of the procedure, and the technical expertise of the surgical team. This lends further support to the notion that hip fractures should ideally be managed expeditiously, by experienced surgeons and anesthetists who are able to complete the surgery safely and quickly.

A previous systematic review found no significant difference between GA and spinal anesthesia on the risk for delirium after hip fracture surgery.^57^ However, our results suggest that GA is associated with a higher incidence of delirium and that prolonged surgical duration is associated with a higher incidence in patients who receive GA. This is consistent with work demonstrating that inhalational anesthetics and propofol can result in decreased cerebral blood flow, particularly in patients with head injuries.^20,58^ Compared with GA, regional blocks allow for appropriate pain control and lower the doses of systemic anesthesia.^59,60^ Animal models also suggest that extended GA may worsen the risk associated with spinal anesthesia,^61,62^ although results in humans have been mixed.^14,63,64^ As such it is plausible that prolonged duration of systemic anesthesia results in an increased risk for delirium. At least 2 multicenter trials are currently underway examining the effect of the type of anesthesia on postoperative delirium in patients with hip fracture.^65,66^

### Strengths and Limitations

This study examines whether duration of surgery is associated with the risk for postoperative delirium for patients who received hip fracture surgery. We were able to examine a large cohort of patients who had a hip fracture from 80 hospitals. In addition, we controlled for various confounders, including patient and hospital factors.

This study had limitations. First, the occurrence of delirium was identified using validated definitions with high specificity but poor sensitivity (35%). It is likely that this definition primarily captures hyperactive delirium, as this is more likely to be identified and documented by physicians and nurses.^37,38,42^ Obviously the degree to which this diagnosis is made varies by center, so we mitigated this limitation by clustering our regression analyses by hospital. The incidence of postoperative delirium in our cohort (approximately 11%) is consistent with previous reports, including the National Institute for Health and Care Excellence guidelines, on the incidence of delirium diagnoses by physicians after hip fracture surgery.^43,44,45^ Second, our regression analysis did not find an association between preexisting dementia and a subsequent diagnosis of delirium, despite evidence that it is a strong risk factor for delirium.^46,47^ We believe this may reflect the nature of the diagnostic code—because this code represents cases of delirium that were diagnoses by the health care team, they likely represent acute changes in patients who had a normal baseline. In patients with a history of dementia, any change consistent with delirium is often attributed to the patient’s baseline function.^48,49^ This is not a fault of the algorithm used to identify postoperative delirium, but represents the limitations of the health care team themselves. The resulting misclassification bias would result in our analysis underestimating the strength of the association between duration of surgery and the risk for postoperative delirium.

A further limitation was our definition of surgical duration, defined as time between entry and exit from the operating room (and not the time between incision and closure, which was not captured). However, these data have high face validity since basic descriptive statistics demonstrate few anomalous observations and sensible surgical durations.^50^ We were also unable to reliably differentiate between incident (postoperative) and prevalent (preoperative) cases of delirium. However, we do not believe that prevalent cases would have an increased duration of surgery or a prolonged induction of anesthesia. There was also no association between the occurrence of delirium preoperatively and the complexity of the fracture.^51^ As such, misclassification between preoperative and postoperative delirium is likely to bias our estimates further toward the null. Our clinical experience suggests that patients with delirium preoperatively often go straight to having GA, and actually have a shorter induction than those who receive a spinal anesthetic.^52^

## Conclusions

Prolonged surgical duration in hip fracture patients was associated with an increased risk for postoperative delirium, as was the use of GA.
